# Anti-Tumor Activity vs. Normal Cell Toxicity: Therapeutic Potential of the Bromotyrosines Aerothionin and Homoaerothionin In Vitro

**DOI:** 10.3390/md18050236

**Published:** 2020-05-01

**Authors:** Antje Drechsel, Jana Helm, Hermann Ehrlich, Snezana Pantovic, Stefan R. Bornstein, Nicole Bechmann

**Affiliations:** 1 Institute of Clinical Chemistry and Laboratory Medicine, University Hospital Carl Gustav Carus, Technische Universität Dresden, Fetscherstrasse 74, 01307 Dresden, Germany; Antje.drechsel@uniklinikum-dresden.de; 2 Department of Medicine III, University Hospital Carl Gustav Carus, Technische Universität Dresden, Fetscherstrasse 74, 01307 Dresden, Germany; jana.helm@uniklinikum-dresden.de (J.H.); Stefan.bornstein@uniklinikum-dresden.de (S.R.B.); 3 Institute of Electronics and Sensor Materials, TU Bergakademie Freiberg, Gustav-Zeuner str. 3, 09599 Freiberg, Germany; hermann.ehrlich@esm.tu-freiberg.de; 4 Center for Advanced Technology, Adam Mickiewicz University, 61614 Poznan, Poland; 5 Department of Medical Biochemistry, Faculty of Medicine, University of Montenegro, Kruševac bb, 81000 Podgorica, Montenegro; snezap@ac.me; 6 Center for Regenerative Therapies Dresden, Technische Universität Dresden, Fetscherstrasse 105, 01307 Dresden, Germany; 7 Department of Experimental Diabetology, German Institute of Human Nutrition Potsdam-Rehbruecke, 14558 Nuthetal, Germany; 8 German Center for Diabetes Research (DZD), 85764 München-Neuherberg, Germany

**Keywords:** marine sponges, *Aplysina cavernicola*, pheochromocytoma and paraganglioma, fibroblasts, spheroids, HUVEC, fractionated treatment, normal tissue toxicity, therapeutic index

## Abstract

Novel strategies to treat cancer effectively without adverse effects on the surrounding normal tissue are urgently needed. Marine sponges provide a natural and renewable source of promising anti-tumor agents. Here, we investigated the anti-tumor activity of Aerothionin and Homoaerothionin, two bromotyrosines isolated from the marine demosponge Aplysina cavernicola, on two mouse pheochromocytoma cells, MPC and MTT. To determine the therapeutic window of these metabolites, we furthermore explored their cytotoxicity on cells of the normal tissue. Both metabolites diminished the viability of the pheochromocytoma cell lines significantly from a concentration of 25 µM under normoxic and hypoxic conditions. Treatment of MPC cells leads moreover to a reduction in the number of proliferating cells. To confirm the anti-tumor activity of these bromotyrosines, 3D-pheochromocytoma cell spheroids were treated with 10 µM of either Aerothionin or Homoaerothionin, resulting in a significant reduction or even complete inhibition of the spheroid growth. Both metabolites reduced viability of normal endothelial cells to a comparable extent at higher micromolar concentration, while the viability of fibroblasts was increased. Our in vitro results show promise for the application of Aerothionin and Homoaerothionin as anti-tumor agents against pheochromocytomas and suggest acceptable toxicity on normal tissue cells.

## 1. Introduction

Cancer is one of the main causes of death in this century and there is a continuing need for the identification and the development of new anti-tumor drugs. The clinical use of new drugs is thereby often limited by their low specificity towards the tumor tissue, leading to simultaneous damage of the surrounding normal tissue and restricting the dose that can be administered (therapeutic window).

Natural resources provide a virtually inexhaustible source of bioactive compounds. Especially the oceans, which cover the majority of our planet, make the marine environment the largest habitat on earth hosting high and largely-unexplored biodiversity [1]. Marine sponges are mostly of sessile nature and lack effective morphological defense mechanisms; nonetheless, their survival is ensured by the development of chemical defense strategies by the production of secondary metabolites with various bioactivities [2,3]. These secondary metabolites have also shown the potential to inhibit tumor cell growth [4,5]. Already six decades ago, isolation of C-nucleosides from the Caribbean sponge, *Cryptotheca crypta*, provided the basis for the synthesis of Cytarabine, the first sponge-derived anti-tumor drug in clinical use [6]. Eribulin (Halaven^®^), a synthetic derivative based on the structure of halichondrin B isolated from the demosponge *Halichondia okadai*, is also in clinical application for the treatment of metastatic breast cancer [4,7]. These data underline the potential of sponge-derived secondary metabolites as anti-tumor drugs.

**Figure 1:**
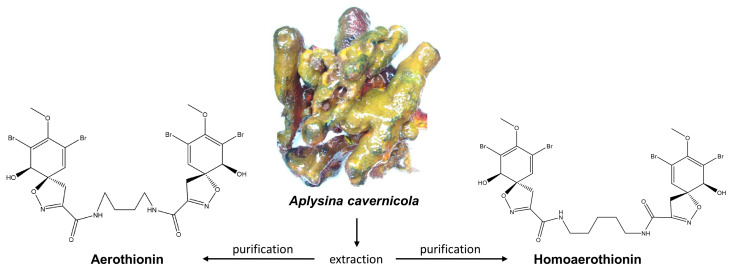
Schematic view: fresh collected *A. cavernicola* demosponge (the diameter of the sponge’s finger-like bodies is about 2 cm) and the chemical structure of two isolated secondary metabolites, Aerothionin and Homoaerothionin.

Sponges of the Verongiida order, such as *Aplysina aerophoba* and *A. cavernicola*, are characterized by the synthesis of brominated tyrosine derivatives (bromotyrosines) with, e.g., cytotoxic and multi-target activities [8]. According to the modern view, bromotyrosines can be produced by spherulocytes—specialized cells located within chitinous skeletal fibers of verongiids [9]. Two corresponding representatives are Aerothionin and Homoaerothionin, two tetrabromo spirocyclohexadienylisoxazoles with a wide range of biological activity (Figure 1) [10]. Aerothionin displayed a cytotoxic activity towards the cervical cancer (HeLa) [11,12] as well as breast cancer (MCF-7) cells [13] and was able to inactivate multidrug-resistant clinical isolates of *Mycobacterium tuberculosis* [14]. Moreover, Aerothionin showed the potential to inhibit the adenosine A1 receptor [12]. Both sponge-derived metabolites inhibit voltage-dependent calcium channels [15] and furthermore showed promising activity against the chloroquine-resistant strain of *Plasmodium falciparum*, the carrier of malaria [13]. Demosponges of the order Verongiida can be cultivated and represent renewable sources of unique *3D* chitinous scaffolds [16,17], which are ready-to-use for various biomedical [18,19] and technological applications [9,20,21,22,23,24].

We previously showed that marine sponges provide a renewable natural source of potential anti-tumor and anti-metastatic drugs for the treatment of adrenal pheochromocytomas and extra-adrenal paragangliomas (PPGLs) [25]. These neural crest-derived tumors with variable disease aggressiveness provide a good model for investigating the anti-tumor activity of new sponge-derived metabolites; in addition, effective treatment strategies for these rare tumors are still lacking [26]. This prompted us to investigate the anti-tumor activity of Aerothionin and Homoaerothionin against pheochromocytoma cells. In this regard, we also evaluate the normal tissue toxicity of both metabolites using fibroblasts and endothelial cells to determine the therapeutic window and to predict undesirable side effects on the normal tissue.

## 2. Results

### 2.1. Anti-Tumor Activity of Aerothionin and Homoaerothionin In Vitro

To evaluate the therapeutic window of Aerothionin and Homoaerothionin, we started with the determination of the anti-tumor activity of both compounds. Therefore, two different pheochromocytoma cell lines were used as models. Tumor cell hypoxia is commonly known to induce therapy resistance. To simulate hypoxic conditions characterized by a reduced oxygen partial pressure, we cultivated both cell lines under extrinsic hypoxia (≤1% O_2_) or spheroid conditions as 3-dimentional model characterized by intrinsic hypoxia [27].

#### 2.1.1. Aerothionin and Homoaerothionin Decreased Proliferating Cell Characteristics

**Figure 2:**
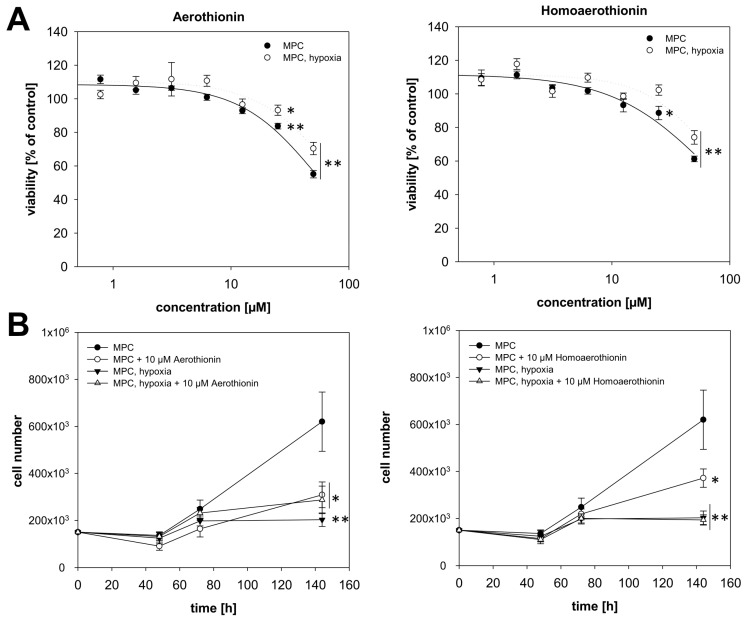
The impact of Aerothionin and Homoaerothionin on MPC cell proliferating properties. The impact of Aerothionin and Homoaerothionin on (**A**) MPC cell viability and (**B**) the number of proliferating MPC cells under normoxic and hypoxic conditions is shown. Four to five independent experiments were performed (n = 15–32). Mean±SEM; ANOVA and Bonferroni post hoc test comparison vs. control * *p* < 0.05, ** *p* < 0.001.

Aerothionin reduced the relative viability (RV) of MPC mouse pheochromocytoma cells after 24 h treatment, significantly starting with a concentration of 25 µM (RV_25µM_ = 83.7 ± 1.7%; RV_50µM_ = 55.1 ± 2.2%; Figure 2A). Under hypoxic conditions, Aerothionin was less effective in decreasing MPC cell viability (RV_25µM_ = 93.2 ± 3.0%; RV_50µM_ = 70.4 ± 3.7%). Treatment with 10 µM Aerothionin diminished the number of proliferating cells under normoxic conditions significantly, while MPC cells under hypoxic conditions were not additionally affected (Figure 2B). As already shown in our previous work, cultivation under hypoxia resulted in growth inhibition of these cells [25].

The second sponge-derived drug, Homoaerothionin, was less effective in diminishing the proliferating properties of these cells. Homoaerothionin reduced RV under normoxic conditions from 25 µM (RV_25µM_ = 88.6 ± 4.0%; RV_50µM_ = 61.3 ± 1.7%), but under hypoxic conditions a concentration of 50 µM (RV_25µM_ = 102.3 ± 3.1%; RV_50µM_ = 74.1 ± 4.0%) was needed to decrease RV significantly (Figure 2A). Under normoxic conditions, treatment with 10 µM Homoaerothionin diminished the number of proliferating MPC cells significantly, while no additional effect could be detected under hypoxic conditions (Figure 2B).

**Figure 3:**
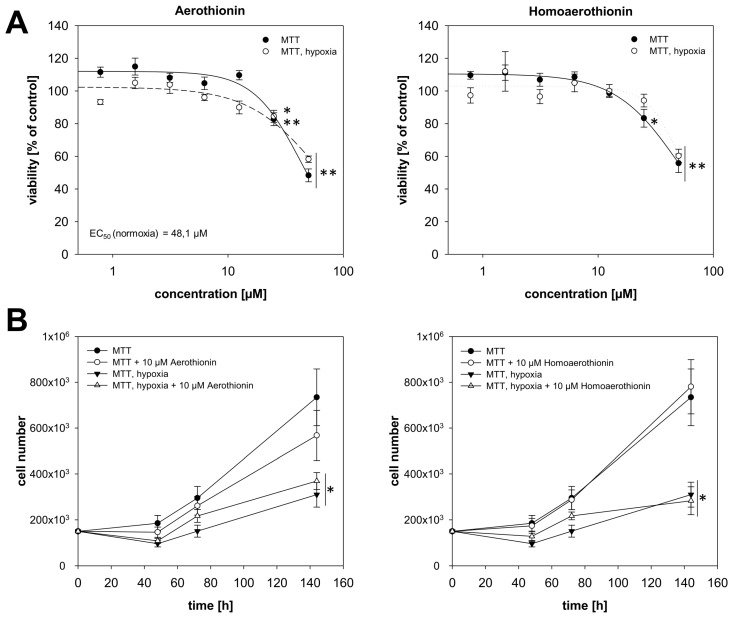
The impact of Aerothionin and Homoaerothionin on MTT cell proliferating properties. The impact of Aerothionin and Homoaerothionin on (**A**) MTT cell viability and (**B**) the number of proliferating MTT cells under normoxic and hypoxic conditions is shown. Four to five independent experiments (n = 15–32). Mean ± SEM; ANOVA and Bonferroni post hoc test comparison vs. control * *p* < 0.05 or ** *p* < 0.001.

For confirmation purposes, we used a second pheochromocytoma cell line, named MTT that shows a more aggressive cell behavior. Treatment with at least 25 µM Aerothionin (RV_25µM_ = 82.8 ± 3.9%; RV_50µM_ = 48.3 ± 4.0%; Figure 3A) diminished RV under normoxic conditions significantly resulting in a half-maximal effective concentration (EC_50_) of 48.1 µM in these cells. Cultivation under hypoxia just slightly impaired the cellular response of the MTT cells towards Aerothionin (RV_25µM_ = 84.5 ± 3.7%; RV_50µM_ = 58.2 ± 2.0%). The number of proliferating MTT cells was not affected by the treatment with Aerothionin under normoxic and hypoxic conditions (Figure 3B). Homoaerothionin reduced the RV under normoxia (RV_25µM_ = 83.3 ± 5.4%; RV_50µM_ = 55.7 ± 5.7%) and hypoxia (RV_25µM_ = 94.2 ± 3.9%; RV_50µM_ = 60.3 ± 4.1%) to a comparable extent (Figure 3A). Similar to Aerothionin, treatment with Homoaerothionin (10 µM) had no effect on the number of proliferating MTT cells (Figure 3B).

#### 2.1.2. Aerothionin and Homoaerothionin Diminished Spheroid Growth

**Figure 4:**
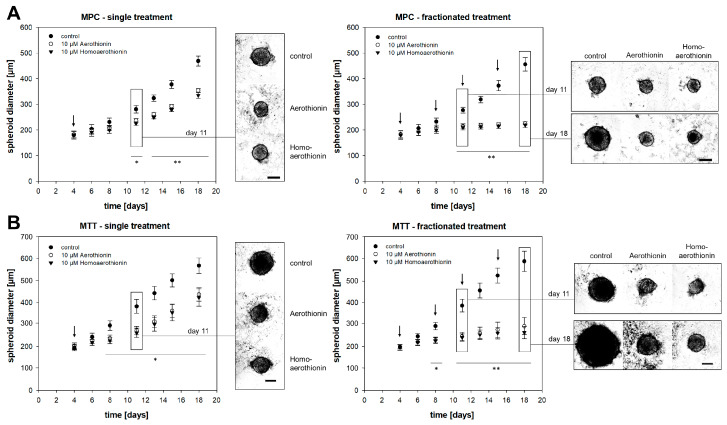
The impact of Aerothionin and Homoaerothionin on pheochromocytoma cell spheroid growth. (**A**) MPC and (**B**) MTT cell spheroids were treated with Aerothionin, Homoaerothionin, or DMSO as the control. A single treatment or a fractionated treatment regime was performed. Arrows mark the different treatment time points. Four independent experiments (n = 12) were performed. Mean ± SEM; ANOVA and Bonferroni post hoc test comparison vs. control * *p* < 0.05, ** *p* < 0.001. Scale bar: 200 µm.

For a better simulation of the in vivo tumor situation, we used *3D*-pheochromocytoma cell spheroids characterized by an oxygen and nutrient gradient that led to the formation of three different zones: (1) the outer layer with proliferating cells; (2) a hypoxic area; and (3) the necrotic core [27]. A single treatment with 10 µM Aerothionin or Homoaerothionin at day four after spheroid generation decelerated MPC cell spheroid growth from day 11 onwards significantly (Figure 4A). In clinical treatment regimes, drugs are often administered in recurring cycles. Therefore, we treated our MPC cell spheroids at days four, eight, 11, and 15 after spheroid generation, and monitored their growth. In contrast to the single treatment, a fractionated treatment regime with Aerothionin or Homoaerothionin resulted in complete inhibition of the MPC cell spheroid growth (Figure 4A). Comparable results were also obtained for the more aggressive MTT cell spheroids characterized in this model by a larger spheroid diameter (diameter_day18_ = 568.0 ± 34.8 µm) compared to the MPC cell spheroids (diameter_day18_ = 469.1 ± 19.5 µm). Four days after single treatment with either Aerothionin or Homoaerothionin the MTT spheroid growth diminished significantly and a fractionated treatment inhibited the growth completely over the observation time.

### 2.2. Effects of Aerothionin and Homoaerothionin on Cells of the Normal Tissue

For the development of a novel therapeutic strategy, the therapeutic window of a drug is of great importance. It is defined as the range of drug dosages that can treat disease effectively without having side effects mainly associated with a toxicity on cells of the normal tissue. Therefore, we investigated the therapeutic effective concentration of 10 µM Aerothionin and Homoaerothionin (anti-tumor activity) regarding a possible cytotoxic effect on endothelial cells and fibroblasts of normal tissue.

#### 2.2.1. Effects of Aerothionin and Homoaerothionin on Endothelial Cells

**Figure 5:**
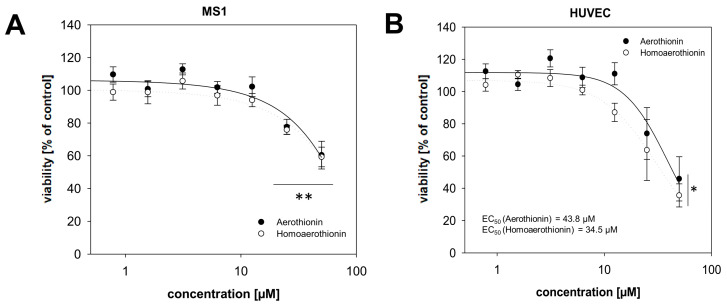
The effects of Aerothionin and Homoaerothionin on endothelial cell viability. Dose-dependent effects of Aerothionin and Homoaerothionin on the cell viability of (**A**) mouse endothelial cell line, MS1, and (**B**) primary human umbilical vein endothelial cells (HUVECs) are shown. Four independent experiments (n = 12) were conducted. Mean ± SEM; ANOVA and Bonferroni post hoc test comparison vs. control * *p* < 0.05, ** *p* < 0.001.

Treatment with 25 to 50 µM Aerothionin (RV_25µM_ = 77.7 ± 4.6%; RV_50µM_ = 60.4 ± 8.5%) or Homoaerothionin (RV_25µM_ = 75.9 ± 2.8%; RV_50µM_ = 59.2 ± 6.0%) significantly reduced the viability of mouse endothelial cells (MS1) isolated from the islet of Langerhans from the pancreas (Figure 5A). The cytotoxic effect of both compounds was thereby comparable. Generation of MS1 cells spheroids was not successful to study long-time effects of Aerothionin and Homoaerothionin. To confirm these results, we used primary human umbilical vein endothelial cells (HUVECs) [28]. Aerothionin (RV_25µM_ = 74.0 ± 16.1%; RV_50µM_ = 45.8 ± 13.7%) or Homoaerothionin (RV_25µM_ = 63.7 ± 18.9%; RV_50µM_ = 35.5 ± 7.3%) diminished the viability of these cells at a concentration of 50 µM (Figure 5B). The half-maximal effective concentration was determined in the middle micromolar range (EC_50,Aerothionin_ = 43.8 µM; EC_50,Homoaerothionin_ = 34.5 µM) for the HUVECs.

#### 2.2.2. Aerothionin and Homoaerothionin Stimulated the Viability of Normal Fibroblasts

**Figure 6:**
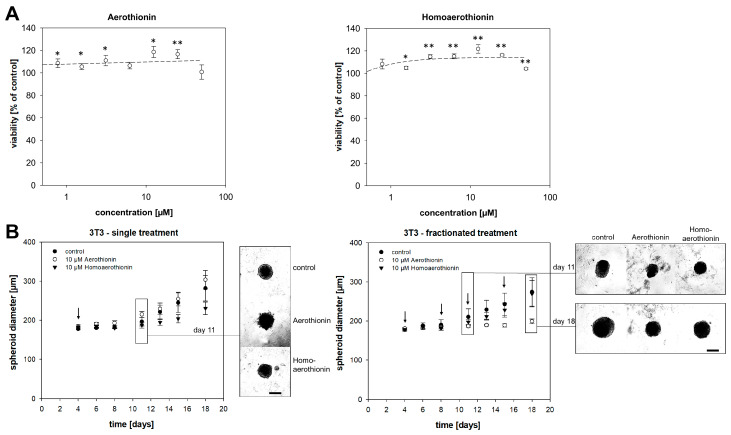
The impact of Aerothionin and Homoaerothionin on fibroblasts. (**A**) Treatment with either Aerothionin or Homoaerothionin stimulated the viability of the mouse fibroblast, 3T3. (**B**) 3T3 fibroblast spheroids were treated with Aerothionin, Homoaerothioin, or DMSO as the control. A single treatment or a fractionated treatment regime was performed. Arrows mark the different treatment time points. Scale bar: 200 µm. Four independent experiments (n = 12) were conducted. Mean ± SEM; ANOVA and Bonferroni post hoc test comparison vs. control * *p* < 0.05, ** *p* < 0.001. No significant difference was verified for 3T3 spheroids.

As a second cell type of the normal tissue, we investigated the effect of Aerothionin and Homoaerothionin on mouse fibroblasts, 3T3. Fibroblasts are crucial for synthesizing the structural framework of tissues. Treatment with up to a concentration of 25 µM Aerothionin (RV_25µM_ = 116.6 ± 4.0%; RV_50µM_ = 100.8 ± 6.4%) stimulated the viability of 3T3 cells significantly (Figure 6A). Homoaerothionin even showed this stimulating effect on 3T3 viability up to a concentration of 50 µM (RV_25µM_ = 116.4 ± 0.8%; RV_50µM_ = 102.3 ± 1.0%). To investigate long-time effects, we treat 3T3 cell spheroids with 10 µM Aerothionin or Homoaerothionin (Figure 6B). The same concentration of Aerothionin or Homoaerothionin, that resulted before in a significant reduction or even a complete inhibition of the tumor cell spheroid growth, showed no measurable effect on the 3T3 spheroid growth after single and fractionated treatment.

## 3. Discussion

The development of novel therapeutic approaches to cure cancer without side effects on healthy tissues is urgently needed. In our present study, we investigated the therapeutic window of two secondary metabolites, Aerothionin and Homoaerothionin, isolated from the marine demosponge *A. cavernicola* for the first time. Both metabolites showed a significant anti-tumor activity towards pheochromocytoma cells, while cells of the normal tissue were either stimulated (in the case of fibroblasts) or reacted with a reduction of viability at higher concentrations (in the case of endothelial cells).

Previously, we have already demonstrated the anti-tumor and anti-metastatic activity of Aeroplysinin-1, a secondary metabolite isolated from the marine demosponge *A. aerophoba*, in our pheochromocytoma cell models [25], and furthermore investigated the anti-tumor activity in cells of more common tumor entities such as melanoma and breast cancer [29]. Aeroplysinin-1 diminished the cell viability of MPC and MTT cells in a micromolar concentration (EC_50,Aeroplysinin-1_ = 9.6–11.4 µM) [25], while Aerothionin and Homoaerothionin reduced the viability of these cells only from a higher concentration of 25 µM. All three secondary metabolites reduced or even inhibited the pheochromocytoma spheroid growth to a comparable extent. This is furthermore in line with the anti-tumor effects of Aerothionin on HeLa cells in a micromolar range [11,12]. In the literature, it is discussed whether Aerothionin and Homoaerothionin are only weakly active precursors, which are converted into the active form, Aeroplysinin-1, by an enzymatic biotransformation following the breakdown of the cellular compartmentation of the sponge [30,31]. Another reason for the slightly diminished anti-tumor activity of Aerothionin and Homoaerothionin might be thereduced availability of these compounds in the cells. Structural differences in comparison to Aeroplysinin-1 indicate a higher hydrophobicity of Aerothionin and Homoaerothionin, possibly leading to a stronger accumulation of these compounds in the hydrophobic cell membrane.

The clinical application of novel drugs is often limited by their unspecific toxicity towards the normal tissue and the associated short- and long-term side effects. At high concentrations of 25–50 µM, Aerothionin and Homoaerothionin diminished viability of mouse (MS1) and primary human (HUVEC) endothelial cells. An EC_50_ of 43.8 µM for Aerothionin and 34.5 µM for Homoaerothionin could only be determined for the HUVECs. Aeroplysinin-1 showed increased cytotoxicity on the MS1 cell (EC_50_ = 18.1 µM) [29], while for Aerothionin and Homoaerothionin no EC_50_ value could be detected. With regard to undesirable toxicity towards the endothelium, Aerothionin and Homoaerothionin appear to be safer than Aeroplysinin-1, with a comparable anti-tumor activity in vitro. Inhibition of tumor angiogenesis is also discussed as a promising therapeutic approach to treat cancer [32]. Nothing is known about the anti-angiogenic activity of Aerothionin and Homoaerothionin, but the anti-angiogenic activity of Aeroplysinin-1, which reduces the growth, migration, and invasion of endothelial cells [33] is well described and also suggests an anti-angiogenetic activity of Aerothionin and Homoaerothionin.

Another cell type of normal tissue is fibroblasts, which are crucial for the formation of the connective tissue and are key players for maintaining skin homeostasis and orchestrating physiological tissue repair [34]. Aerothionin and Homoaerothionin did not show any short-term and long-term cytotoxic effects on fibroblasts; on the contrary, the treatment of both compounds resulted in stimulation of fibroblast viability. In comparison, Aeroplysinin-1 reduced the viability of the 3T3 fibroblasts significantly (EC_50_ = 40.1 µM) [29]. The potential fibroblast stimulating effects of Aerothionin and Homoaerothionin should be investigated further, especially with regard to a potential application to induce wound-healing and tissue renewal [35]. Overall, our in vitro data indicates that Aerothionin and Homoaerothionin have a wider therapeutic window than Aeroplysinin-1, demonstrating a comparable anti-tumor activity with simultaneously-reduced toxicity on cells of normal tissue.

Moreover, dose-limiting side effects on the normal tissue and the potentially limited bioavailability of Aerothionin and Homoaerothionin could, for example, be improved by targeting chemical modification of the molecule structure or an encapsulation of the drug [36,37,38]. We previously demonstrated that a targeted release of nitric oxide could diminish cytotoxic effects on endothelial cells induced by selective estrogen receptor modulators (SERMs) and could thereby improve the therapeutic index of these drugs [38]. The diverse effects on different types of cells indicate a cell-specific target resulting in the anti-tumor activity of these metabolites. Kalaitzis et al. already demonstrated an inhibitory activity of Aerothionin on the adenosine A1 receptor [12]. An effect on voltage-dependent calcium channels was furthermore discussed for both metabolites [15]. Continuative investigations on Aerothionin and Homoaerothionin should therefore focus on the identification of the precise mechanism of action leading to the anti-tumor activity of these bromotyrosines.

In the present study, we demonstrated the anti-tumor activity of Aerothionin and Homoaerothionin on pheochromocytoma cells. For non-metastatic pheochromocytomas and paragangliomas, surgery is the treatment of choice, but if metastases already occur treatment is challenging [39]. Combination therapy with BYL719, a phosphatidylinositol-3-kinase α inhibitor, and everolimus, a mammalian target of rapamycin inhibitor, showed synergistic effects on PPGLs in vitro [40]. The presence of different secondary metabolites in the sponge also implies that the extent of the chemical defense mechanism might be due to the combination of different metabolites. Therefore, it would be interesting for further studies to examine whether Aerothionin and Homoaerothionin might also have a synergistic anti-tumor effect either with other sponge-derived secondary metabolites or common chemotherapeutic agents (chemosensitizing effect).

Our in vitro investigations showed promise for the application of Aerothionin and Homoaerothionin as anti-tumor drugs against PPGLs. The therapeutic application of these precursors of Aeroplysinin-1 seems to have a better therapeutic window than the active compound showing a comparable anti-tumor activity by reduced toxicity on cells of normal tissue. The application of Aerothionin and Homoaerothionin, as well as other sponge-derived secondary metabolites, provides a promising therapeutic approach to treat cancer alone or perhaps also in combination with other drugs.

## 4. Materials and Methods

Aerothionin and Homoaerothionin were kindly provided by BromMarin GmbH (Freiberg, Germany) with the a purity grade of >99%.

### 4.1. Cell Culture

The mouse pheochromocytoma cells (MPC) generated from heterozygous neurofibromatosis knockout mice, and its more aggressive derivate, the tumor tissue-derived (MTT) cells, were acquired from Arthur Tischler [41,42,43] and cultivated as previously described [25]. Mouse fibroblasts 3T3 isolated from embryo tissue and the mouse endothelial cells, Mile sven 1 (MS1), were obtained from the American Type culture collection and cultivated using Dulbecco’s Modified Eagle’s Medium (DMEM) with 10% fetal calf serum (FCS) plus 1 mM glutaMax and sodium pyruvate. Primary human umbilical vein endothelial cells (HUVECs) were isolated and cultivated as previously described [28]. All cells were cultivated under normoxic conditions in a CO_2_ incubator. To simulate hypoxic conditions (extrinsic hypoxia), cells were cultivated at reduced oxygen partial pressure (≤1% O_2_) in a special incubator equipped with an oxygen-sensor (Gasboy, Labotect, Rosdorf, Germany). In all cases, cultivation took place at 37 °C, 5% CO_2_, and 95% humidity. All cell lines were routinely tested to be mycoplasma-free using MycoAlert Mycoplasma Detection Kit (Lonza, Basel, Switzerland). After trypsinization (trypsin/EDTA; 0.05%/0.02%) cells were diluted with complete medium and counted using C-CHIPs (Neubauer improved). All experiments were performed after at least one passage after re-cultivation. In the case of the pheochromocytoma cells, cultivation and the experimental work were performed by using collagen A coated cell culture dishes. For the primary HUVEC cells, cell culture dishes were coated with gelatin (0.5%).

### 4.2. Viability Assay

To investigate the anti-tumor activity of Aerothionin and Homoaerothionin, the CellTiter 96^®^ AQueous One Solution Cell Proliferation Assay (Promega, Walldorf, Germany) was used as previously described [25]. Both compounds were used at a concentration of 0.4 to 50 µM and the relative viability was calculated using the analyzed absorbance at 490 nm ([absorbance_treated_ × 100%]/absorbance_DMSOcontrol_). The half-maximal effective concentration (EC_50_) was calculated from the dose-response curve by using the dose-response fit model of the SigmaPlot software package.

### 4.3. Proliferation Assay

Cells (1.5 × 10^5^) were seeded in 6-well plates, allowed to attach for 24 h and treated with 10 µM Aerothionin and Homoaerothionin. Afterwards, cells were incubated for 48 h, 72 h, or 144 h under normoxic or hypoxic conditions. Cells were washed with PBS, trypsinized, and after careful resuspension in medium (total volume: 1 mL) cells were counted using the cell counting application of the Spark^®^ multimode microplate reader (Tecan Trading AG, Männedorf, Switzerland). Each well was counted in duplicate.

### 4.4. Generation and Cultivation of Tumor Cell Spheroids

Pheochromocytoma cell spheroids were generated as previously described [25,27].

### 4.5. Generation and Cultivation of Fibroblast Spheroids

The 3T3 cells (5 × 10^2^) were resuspended in complete DMEM containing 20% of a 1.2% methylcellulose solution (0.24% (*w*/*v*), prepared in serum-free DMEM + Glutamax) and seeded in non-adherent round-bottom 96-well plates for suspension culture (Greiner Bio-One, Kremsmünster, Austria). After 3–4 days of cultivation, the consumed medium was replaced.

### 4.6. Spheroid Treatment and Growth Measurement

To determine the influence of Aerothionin and Homoaerothionin, four-day-old spheroids were treated with 10 µM of the sponge-derive secondary metabolites. Two different experimental settings were performed. For the first one, treatment took place once at day four (single treatment). During the second setting, spheroids were treated 4, 8, 11, and 15 days after generation (fractionated treatment). Afterward, the size of each spheroid was measured by using an inverse microscope Axiovert 200M (Zeiss, Software: AxioVision 4.8). The area (A) of each spheroid was analyzed using the software package Fiji (ImageJ). The diameter (d) was calculated under the assumption of an approximately spherical form of the spheroids (d = 2 × √(A/π)).

### 4.7. Statistical Analysis

Descriptive data were expressed as mean ± SEM. The number of n represents the number of technical and biological replicates within the independent experiments. Statistical analysis was carried out using one-way analysis of variance with post hoc Bonferroni with SigmaPlot 12.5 (Systat Software GmbH, Erkrath, Germany).
